# Special Issue “Influenza Viruses: Infection and Genomics”

**DOI:** 10.3390/ijms27010506

**Published:** 2026-01-03

**Authors:** Daniele Focosi

**Affiliations:** North-Western Tuscany Blood Bank, Pisa University Hospital, 56124 Pisa, Italy; daniele.focosi@gmail.com

Of the four known influenza virus types affecting humans (A, B, C, D), influenza virus A (IAV) has threatened human health globally for centuries. Decades of genomic studies have increasingly refined the taxonomy of IAV, which now includes subtypes, clades, and genotypes. In this Special Issue of the Journal, titled “*Influenza Viruses: Infection and Genomics*”, several intriguing primary research articles are introduced.

Savin et al., at the Siberian Branch of the Russian Academy of Sciences, used integrative bioinformatics analysis to identify the core genes (*Birc5*, *Cdca3*, *Plk1*, *Tpx2*, *Prc1, Rrm2*, *Nusap1*, *Spag5*, *Top2a*, and *Mcm5*) and transcription factors (*E2F1*, *E2F4*, *NF-YA*, *NF-YB*, and *NF-YC*) involved in persistent lung injury and regeneration processes in IAV-infected murine lung tissue. Further in vivo verification of the core signature genes confirmed their involvement not only in IAV infection but also in COVID-19 and lung neoplasm development, suggesting their potential role in abnormal epithelial proliferation and oncotransformation [Contribution 1].

Yin et al., at the Nanjing Agricultural University, reported an increased expression of Linc01615, a long intergenic non-coding RNA (LincRNA), in A549 cells upon IAV PR8 infection. The authors proved that DHX9, a protein interaction predicted by the catRAPID website, binds with Linc01615 to partake in IAV replication and that Linc01615 helps to activate the intracellular immune system, which was confirmed by knockdown experiments and by cross-linking immunoprecipitation and high-throughput sequencing (CLIP-seq) experiments [Contribution 2].

Hao et al., at the University of Texas Health Science Center, reported that upon H_1_N_1_ IAV infection, Runx3 promoted enlargement of mediastinal lymph nodes (mLNs) and enhanced CD8^+^ and CD4^+^ T cell expansion in lung-draining mLNs but not in lungs. Runx3 is required for CD43 core 2 O-glycosylation on activated CD8^+^ T lymphocytes, and the involved Runx3 signal pathway may mediate the CD8^+^ T lymphocyte phenotype for generation of pulmonary CTLs [Contribution 3].

Savenkova et al., at the Russian State Research Center of Virology and Biotechnology “Vector”, reported that in mice, the knockout of the tumor necrosis factor alpha (TNF-α), a well-known proinflammatory cytokine, leads to increased viral loads compared to the parental strain but similar amounts of live virus and lower interalveolar septal infiltration [Contribution 4].

IAV(H_5_N_1_) clade 2.3.4.4b is becoming enzootic in mammals and reinforces its position as a pandemic candidate. This Special Issue finally includes a review on 2.3.4.4b, in particular genotype B3.13, which has recently caused an outbreak in US dairy cattle. Since pandemic preparedness is largely based on the availability of prepandemic candidate vaccine viruses (CVVs), we have reviewed challenges for H_5_ vaccine manufacturing and delivery [Contribution 4]. In this regard, mRNA vaccines represent the most promising pipeline in terms of turnaround times and combination.

Much remains to be discovered about IAVs. The exact subtype and clade that will cause the next pandemic remain difficult to predict, with a long list of candidate subtypes circulating at low levels across birds and mammals and occasionally causing outbreaks in humans exposed for professional reasons. Much research is still needed to precisely identify host restriction factors. While we have gained some insights into serological cross-reactivities and hence into candidate vaccine virus (CVV) efficacy, under pre-pandemic scenarios, the ability to maintain a robust veterinary genomic surveillance program will be fundamental to facilitate drug manufacturers, including those dealing with passive immunotherapies. Similarly, the ability to manufacture combined vaccinations could boost the otherwise declining vaccine compliance rates. Moreover, concerns persist regarding the real-world clinical efficacy of small-molecule antivirals and the risk of mutational escape during massive deployments. As such, preparedness should include frameworks for immediately starting well-designed randomized clinical trials.

